# Emergency department crowding and hospital transformation during COVID-19, a retrospective, descriptive study of a university hospital in Stockholm, Sweden

**DOI:** 10.1186/s13049-020-00799-6

**Published:** 2020-10-28

**Authors:** Björn af Ugglas, Niclas Skyttberg, Andreas Wladis, Therese Djärv, Martin J. Holzmann

**Affiliations:** 1 Theme of Emergency and Reparative Medicine, Karolinska University Hospital, 141 86 Stockholm, Sweden; 2 Department of Medicine, Solna, Karolinska Institutet, 171 77 Stockholm, Sweden; 3 Department of Medical Informatics, Karolinska University Hospital, 141 86 Stockholm, Sweden; 4 Department of Learning, Informatics, Management and Ethics, Karolinska Institutet, 171 77 Stockholm, Sweden; 5 Division of Surgery, Orthopaedics and Oncology, Linköping University Hospital, 581 85 Linköping, Sweden; 6 Department of Biomedical and Clinical Sciences, Linköping University, 581 83 Linköping, Sweden

**Keywords:** Emergency department, Crowding, COVID-19, Surge capacity, Bed occupancy

## Abstract

**Objectives:**

COVID-19 presents challenges to the emergency care system that could lead to emergency department (ED) crowding. The Huddinge site at the Karolinska university hospital (KH) responded through a rapid transformation of inpatient care capacity together with changing working methods in the ED. The aim is to describe the KH response to the COVID-19 crisis, and how ED crowding, and important input, throughput and output factors for ED crowding developed at KH during a 30-day baseline period followed by the first 60 days of the COVID-19 outbreak in Stockholm Region.

**Methods:**

Different phases in the development of the crisis were described and identified retrospectively based on major events that changed the conditions for the ED. Results were presented for each phase separately. The outcome ED length of stay (ED LOS) was calculated with mean and 95% confidence intervals. Input, throughput, output and demographic factors were described using distributions, proportions and means. Pearson correlation between ED LOS and emergency ward occupancy by phase was estimated with 95% confidence interval.

**Results:**

As new working methods were introduced between phase 2 and 3, ED LOS declined from mean (95% CI) 386 (373–399) minutes to 307 (297–317). Imaging proportion was reduced from 29 to 18% and admission rate increased from 34 to 43%. Correlation (95% CI) between emergency ward occupancy and ED LOS by phase was 0.94 (0.55–0.99).

**Conclusions:**

It is possible to avoid ED crowding, even during extreme and quickly changing conditions by leveraging previously known input, throughput and output factors. One key factor was the change in working methods in the ED with higher competence, less diagnostics and increased focus on rapid clinical admission decisions. Another important factor was the reduction in bed occupancy in emergency wards that enabled a timely admission to inpatient care. A key limitation was the retrospective study design.

## Introduction

### Background

The COVID-19 outbreak presents large challenges to the emergency care system. The underlying SARS-CoV-2 virus appears highly contagious to both staff and other patients and the resultant disease is potentially deadly, especially for older patients with underlying comorbidities such as active cancer, obesity, coronary artery disease, diabetes and chronic obstructive pulmonary disease [1]. The progress of the disease for patients that require intensive-care can be quick with acute respiratory distress, severe hypoxia, acute renal failure and rapidly deteriorating vital parameters [2]. Altogether this results in challenges for the emergency department (ED) [3–5] as patients with suspected COVID-19 needs to be separated from other patients, the staff have to wear protective gear that limit productivity, and vital parameters needs to be re-evaluated with high frequency.

There is a high risk that this increased workload could lead to ED crowding [6] that is known to have a negative impact on patient outcomes [7–9] and the working environment for the staff [10]. A key characteristics of crowding is that when demand exceeds capacity, queues are formed in various part of the system which leads to an extended average ED length of stay (ED LOS) [11–14]. The emergency care system is complex and it is helpful to use the conceptual model introduced by Asplin et al. that partitions the emergency care system in three main components: input, throughput, and output [15]. The input component includes factors that “impact the demand for ED services”. The throughput component is focused on ED internal care processes including triage, physician assessment, diagnostics and treatment. The output component is focused on factors related to the patient disposition and the ability to timely admit patients to inpatient care or safely discharge to outpatient- or self-care.

## Methods

### Aim

The aim is to describe how the Huddinge site at the Karolinska university hospital (KH) responded to the COVID-19 crisis, and how ED crowding, and important input, throughput and output factors for ED crowding developed at KH during a 30-day baseline period followed by the first 60 days of the COVID-19 outbreak in Stockholm Region.

### Study design

**Fig. 1:**
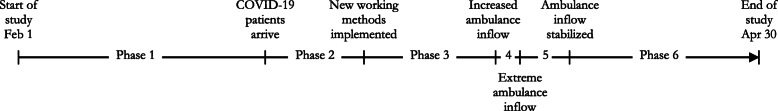
Overview of major events and the six phases in the study. Timeline with major events, defining the transition between the six phases in the development during the 90-day study period

This is a retrospective descriptive study of KH response to the COVID-19 crisis. In the study, the development of the crisis was divided into six different phases separated by five major events that changed the conditions for the ED (Fig. 1). The five major events were defined retrospectively by the research team. The first event was when patients with COVID-19 started to arrive at the ED. The next major event was the implementation of new working methods to cope with this. The last three events were based on sudden changes in the inflow of ambulances due to ambulance diversion from other hospitals following regional decisions. ED crowding together with important input, throughput and output factors for ED crowding were studied for each phase using existing data from the hospital data warehouse.

### Study setting

KH is the southern site at Karolinska university hospital. KH’s assignment is to deliver emergency care, specialized and in some areas highly specialized care in combination with research and education. KH has 760 beds and the ED had 53,508 visits in 2019. The ED has a low proportion of non-urgent and non-complex patients as these will be sorted to a co-located ED with imaging capability led by general practitioners. The department for infectious diseases at KH is the largest in the country and the hospital is the primary receiver of patients with suspected highly infectious diseases in the region. The department for perioperative medicine and intensive care is organized in a single organization responsible for intensive care and the operating theatres supporting all surgical specialties with perioperative care. A brand-new operating theatre with 23 operating rooms was just completed at KH and was meant to be inaugurated during the study period. The department of Emergency and Internal Medicine manage the ED and the emergency wards. In the last few years there has been a growing focus on emergency medicine at KH and there are currently 30 emergency medicine residents in training to become specialists. The ED is mainly staffed with physicians from this department and the ED is divided into medical and surgical patient flows. There is a long-term trend at KH with increased ED crowding and boarding. Unpublished data from standard internal reports show that the mean ED LOS for admitted patients increased by 55% from 4.4 h in the year 2013 to 6.8 h in 2019.

### Detailed description of the six phases in the study

#### Phase 1, “baseline” (Feb 1 to Mar 1)

This phase begins at the start of the study and ends after the winter school holiday. This phase represents a situation where no, or very few suspected COVID-19 patients arrived in the ED.

#### Phase 2, “Early phase, normal working methods” (Mar 2 – Mar 13)

This phase starts when many in the population of Stockholm return from the winter school holiday. Many families returned from travels to countries where the disease was already widely disseminated, which accelerated the spread of the disease in the Stockholm Region. The following week on March 9, a regional decision confirmed that all patients with suspected COVID-19 should be directed to KH. According to standard protocol, patients were received in an isolation room where investigations would be completed before it was decided whether to discharge or admit the patient. After a few days, it became evident that this process would not cope with the volumes of patients and new routines and practices were developed. On March 12 at 14:00, the hospital raised the alertness level to level two out of three according to the Hospital Emergency Operations Plan. This means a partial mobilization of hospital resources and includes cancelling all planned treatments that can wait and the establishment of a Hospital Command Group [16] working according to European/NATO guidelines [17]. During this phase there was a ramp-up of the regional call-center “1177” giving advice on when and where to seek care and the population in Stockholm was asked to always call before seeking care. An online tool for self-triage was also launched. Guidelines for seeking care at the ED’s were strict and patients that did not experience rapid deterioration or had respiratory disorders at rest were directed to self-care [18].

#### Phase 3, “Intermediary phase, new working methods” (Mar 14 – Mar 29)

On March 14, major changes in routines and practices were implemented. These were fine-tuned during the following phases using an agile approach with high presence from first-line management. All patients were now received in a tent outside the ED staffed by at least a resident physician in emergency medicine, an experienced nurse together with a nurse assistant. An initial assessment was performed, and patients were sorted depending on if COVID-19 was suspected or not. Patients with suspected COVID-19 that had mild symptoms and did not belong to a high-risk group were referred to the co-located GP-led ED or diverted to self-care and isolation at home with instructions if the situation deteriorated. Inside, the ED was separated into two equally sized sections where patients with suspected COVID-19 were sorted to one section while all other patients were sorted to the other section. The exception was orthopaedic patients that were now treated in the elective department during office hours. The inflow was initially much lower in the COVID-19 section, while the other section struggled with maintaining a high patient flow, now with half the number of available ED rooms and fewer resources in relation to the number of patients. Competence in the ED was strengthened as all residents in emergency medicine where called back from external rotations and replaced interns that were instead transferred to the emergency wards. Staffing was reinforced during evenings and nights and the surgical specialist position was now staffed 24/7 instead of only weekdays 10–18. Practices were also changed, after an initial assessment and work-up of unstable patients, an early decision on admission to inpatient care was made based on basic lab tests, point-of-care blood gases, and the overall clinical picture. Imaging and further diagnostics were kept to a minimum. The early decision to quickly admit patients with suspected COVID-19 to inpatient care without complete diagnostics was enabled by the transformation of one of the emergency wards with 22 single rooms that performed further investigation and diagnostics that in normal cases would have been performed in the ED. As soon as a patient had a result on their COVID-19 test they would be transferred to an infection ward if positive and to another ward if negative unless they were stable enough to be discharged home. During this phase, the hospital increased the number of beds in the infection wards and started the ramp-up of intensive care capacity. KH was also no longer the primary receiver in the region as in phase 2 and the inflow of patients arriving with ambulance was shared among all of seven hospitals in the region based on geographical segmentation.

#### Phase 4, “Exponential phase with controlled ambulance inflow” (Mar 30 – Apr 2)

This phase starts with the implementation of a regional decision to rebalance the load across the seven hospitals in the region. In the earlier phases, ambulances were directed to the closest hospital, but clustered outbreaks in some of the suburbs led to a congestion of COVID-19 patients in three of the sevens hospitals in the region. The decision led to an increased inflow of ambulances at KH. During this period, the intensive care capacity was further increased at KH to manage the increased inflow of critically ill patients.

#### Phase 5, “Exponential phase with extreme ambulance inflow” (Apr 3 – Apr 8)

This phase starts with a regional decision of directing all ambulances with patients showing respiratory symptoms in Region Stockholm towards KH. The rapid build-up of intensive care capacity in KH now enabled the hospital to relieve the other hospitals in the region to handle about 50% of the regional projected patient flow for intensive care. Even if there was now available intensive care capacity in the hospital, the large inflow of critically ill patients during a short time-period was extremely challenging for the ED. Due to the impact that the first decision had on KH, a new regional decision was taken April 5 that reduced the ambulance diversion for all priority 1 ambulances, and all ambulances from two of the six other hospitals.

#### Phase 6, “Plateau, stabilization of inflow” (Apr 8 – Apr 30)

This phase starts with a regional decision to further reduce the ambulance inflow to KH back to the level in phase 4 again. The number of new cases and patients admitted into intensive care in the Stockholm region also peaked and reached a plateau at this time [19]. Intensive care capacity was now increased to its planned maximum capacity. During this phase there was a shift in patient flows where many patients who had been cared for in the intensive care units had recovered enough to come out of the ventilator but were still very weak, many of them suffering from hypoxemia. To care for post-intensive care patients, the capacity of the high-dependency emergency care unit was increased gradually from 6 to 18 beds by closing down other emergency care wards and relocate the staff. This increased the level of care but reduced the number of available beds.

### Variables

As a proxy for the outcome ED crowding we used the mean ED LOS for each phase. Factors potentially impacting crowding were grouped into input, throughput and output. Input factors were the number of patient visits and the number of patient visits arriving by emergency medical services (EMS). Throughput factors indicating the share of diagnostics and treatment performed in the ED were the proportion of patient visits that included medical imaging during the ED stay and the proportion of patient visits that resulted in an admission to inpatient care. Output factors were the bed occupancy level in the emergency wards and the number of care episodes at the emergency wards, together indicating the available capacity for the ED to admit patients to inpatient care without a finalized diagnosis. Another output factor was the total hospital bed capacity indicating the general ability to admit patients to inpatient care. To provide a view on this, the average number of staffed inpatient beds together with the average number of patients by type of ward, phase and date were presented. The wards were grouped in four categories: intensive care, emergency care, infection, and other wards (excluding pediatric and obstetric wards). Basic demographic information on the distribution of sex and age in intervals < 40, 40–59, 60–79 and 80+ were also presented.

### Data sources/measurement

Data used in the study was based on statistics extracted from KH data warehouse. Crosstabs of data were delivered to the research team using Tableau Desktop 2018.3.0 and imported into RStudio 1.1.463 and R version 3.6.1 to create the table, the analysis of correlations and high-resolution graphs. ED LOS is measured from the first registration at the front desk or in the later stages in the tent outside the ED, until the patient leaves the ED. Bed occupancy is measured as the daily average of observations on number of staffed inpatient beds and number of admitted patients that is registered at each ward. The number of staffed inpatient beds is entered manually by each ward as soon as the status changes. The number of patients in each ward is updated automatically as patients are admitted and discharged in the electronic healthcare records. The Data warehouse stores information on beds and patients with 15 min resolution so each daily average is based on 96 observations.

### Statistical methods

For the outcome ED crowding, mean ED LOS was estimated with 95% confidence interval for each phase. Input, throughput and output factors previously known to impact ED crowding together with age and sex were described for each phase using distributions, means and proportions. Pearson correlations with *P*-value and 95% confidence intervals where each phase represented a single observation were estimated between ED LOS and the key input, throughput and output factors. The input factor used was average visits per day, the throughput factor was proportion of imaging performed and the output factor was bed occupancy in the emergency wards. Development of staffed inpatient beds and average number of patients were visualized in line graphs by the type of ward, phase and date.

## Results

### Participants

In total there were 9754 ED visits during the 90-day study-period from February 1 to April 30. Three hundred ninety four visits (4%) were excluded due to missing triage priority while 13 visits (0%) were excluded due to missing information on sex or ED LOS. In total, 9347 visits (96%) were included.

### Input factors

Visits per day were similar in the baseline phase 1 (118) and phase 2 (114) but declined in phase 3 (93). In phase 4 and 5 the number of visits increased to 103 and 123. This was mainly due to an increase in the number of EMS arrivals that reached a maximum of 75 in phase 5 compared to 43 in the baseline phase 1. The proportion of EMS arrivals increased to 49% in phase 4 and 61% in phase 5 compared to 37% in the baseline phase 1.

### Throughput factors

In phase 3, the proportion of patients that had some kind of imaging performed during their ED visit dropped to 18% compared to 32% in the baseline phase 1 and then further to 15 and 11% in the following two phases. Admission to inpatient care developed in the opposite direction with an increase to 43% in phase 3, 48% in phase 4 and 51% in phase 5 compared to 33% in the baseline phase 1 (Table 1).

**Table 1:** Patient visit information by phase

	Phase 1	Phase 2	Phase 3	Phase 4	Phase 5	Phase 6
**Input**						
Visits						
Visits, n	3546	1368	1482	410	613	1928
Days, n	30	12	16	4	5	23
Visits per day, n	118	114	93	103	123	84
EMS arrival						
Arrivals per day, n	43	40	36	50	75	47
Proportion, %	37%	35%	39%	49%	61%	56%
**Throughput**						
Imaging performed						
Yes, %	32%	29%	18%	15%	11%	19%
Admitted to inpatient care						
Yes, %	33%	34%	43%	48%	51%	46%
**Output**						
Emergency ward						
Bed occupancy, %	90%	85%	66%	71%	79%	70%
Care episodes per day, n	27	31	35	33	42	23
Length of stay, hours	50	48	26	23	24	27
**Demographics**						
Sex						
Male sex, n (%)	1686 (48%)	677 (50%)	749 (51%)	213 (52%)	328 (54%)	1036 (54%)
Age						
< 40, n (%)	975 (28%)	392 (29%)	416 (29%)	95 (24%)	138 (23%)	461 (24%)
40–59, n (%)	897 (26%)	385 (29%)	439 (30%)	138 (35%)	206 (34%)	568 (30%)
60–79, n (%)	1074 (31%)	398 (30%)	438 (30%)	119 (30%)	161 (27%)	598 (32%)
80+, n (%)	515 (15%)	165 (12%)	158 (11%)	46 (12%)	96 (16%)	266 (14%)
Missing, n	85	28	31	12	12	35
**ED crowding**						
ED LOS						
Mean, minutes	440	386	307	341	351	294
Confidence interval, 95%	431–449	373–399	297–317	321–360	336–366	287–302

Patient visit information for 9754 ED visit during 90 days from February 1 to April 30 in the Huddinge site of Karolinska University hospital. Variables are grouped in input, throughput and output factors, and by phase in the development

*EMS* Emergency medical services (ambulance or helicopter), *ED LOS* Emergency department length of stay

### Output factors

Mean bed occupancy in the emergency wards declined to 66% in phase 3 compare to 90% in the baseline phase 1. This was followed by an increase to 71% in phase 4 and 79% in phase 5 before it declined to 70% in phase 6. Emergency ward production measured as the mean number of completed care episodes per day increased during phase 2–5 as the lower bed occupancy was offset by a shorter length of stay in the emergency ward (Table 1).

**Fig. 2:**
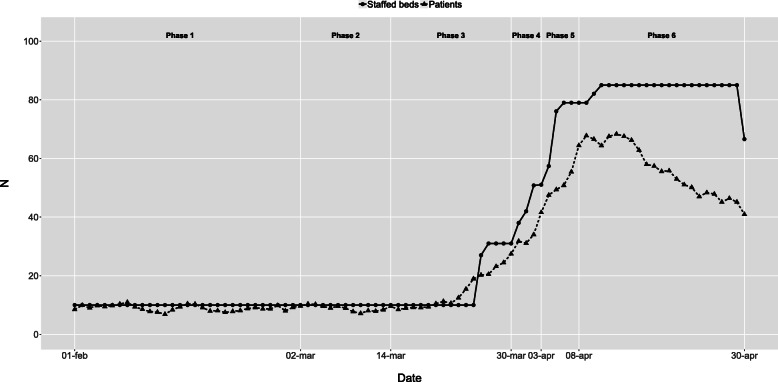
Intensive care wards - Staffed beds and patients by phase and date. Staffed inpatient beds and number of patients in intensive care wards by date and phase

**Fig. 3:**
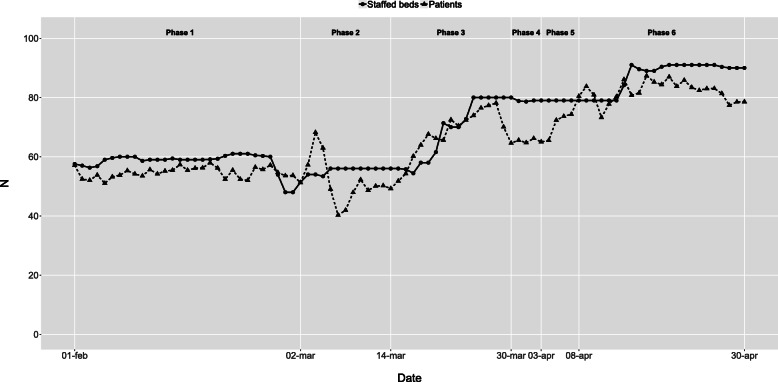
Infection wards - Staffed beds and patients by phase and date. Staffed inpatient beds and number of patients in infection wards by date and phase

**Fig. 4:**
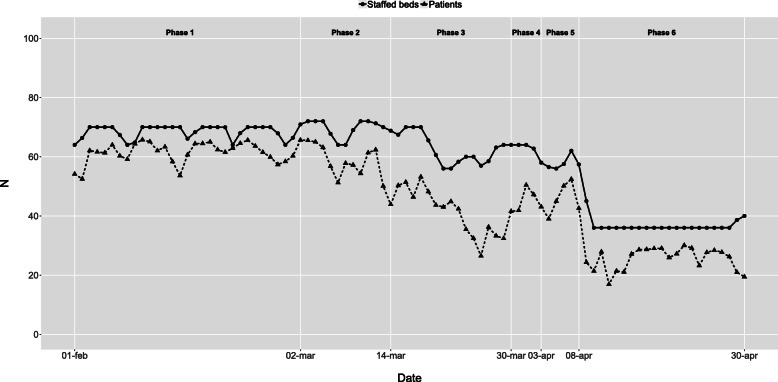
Emergency wards - Staffed beds and patients by phase and date. Staffed inpatient beds and number of patients in emergency wards by date and phase

**Fig. 5:**
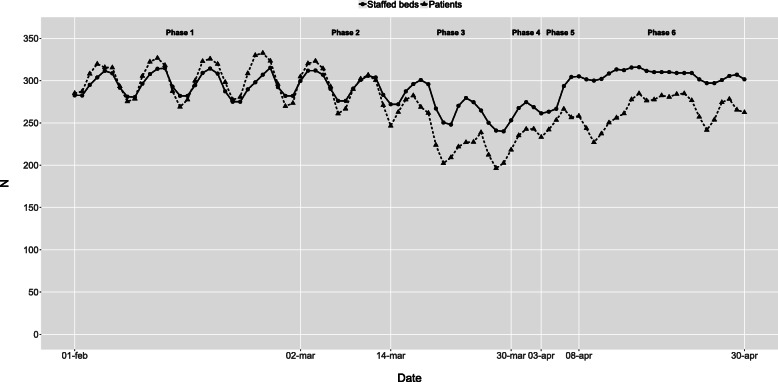
Other wards - Staffed beds and patients by phase and date. Staffed inpatient beds and number of patients in other wards by date and phase

The initial intensive care capacity was 10 staffed beds in the baseline phase 1. In phase 3, the extreme expansion started, resulting in a maximum of 85 staffed intensive care beds during most of phase 6 (Fig. 2). Infection ward capacity had a similar but not as dramatic development (Fig. 3). Emergency wards had an initial capacity of around 70 staffed beds in the baseline phase 1. In phase 3, the average number of patients declined, and in phase 6, the number of beds was reduced to 36 as the normal emergency wards were closed and the staff were relocated to the more densely staffed high-dependency emergency ward (Fig. 4). The capacity of the remaining wards was around 300 staffed beds during the baseline phase 1. Bed capacity declined during phase 2–3 as staff were reallocated to the emergency, infection and intensive care wards. In phase 5, the capacity increased back to 300 again, but the cyclical weekly variation with lower capacity during the weekends disappeared as wards were refocused from elective care towards caring for COVID-19 or other emergency care patients 24/7 (Fig. 5).

### Outcome

The mean (95% CI) ED LOS was 440 (431–449) minutes at the baseline phase 1 and declined to 386 (373–399) in phase 2 and 307 (297–317) in phase 3. It then increased to 341 (321–360) in phase 4 and 351 (336–366) in phase 5 before again declining to 294 (287–302) minutes in the final phase 6. All phases following the implementation of new working methods in phase 3 had a statistically significant lower mean ED LOS than both the first two phases (Table 1).

### Correlation between key factors and ED LOS by phase

**Fig. 6:**
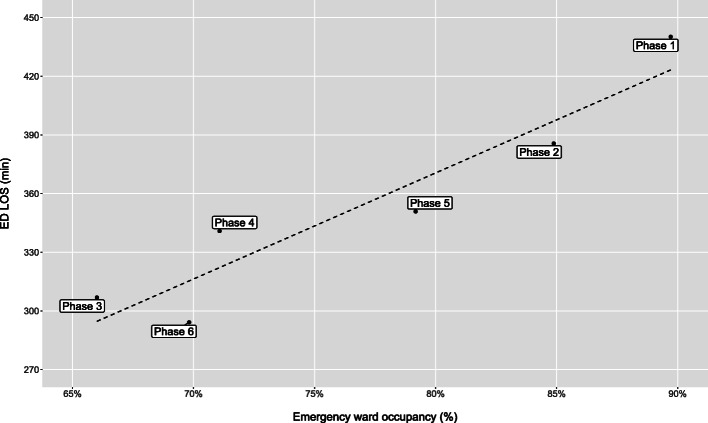
ED LOS by Emergency ward occupancy. Emergency department length of stay (ED LOS) by emergency ward occupancy by phase. Each phase is represented as one observation in the scatterplot

Pearson correlation (95% CI) between mean ED LOS and mean emergency ward bed occupancy for each phase was 0.94 (0.55–0.99) with *P* = 0.005 (Fig. 6). Results for the other factors were not statistically significant with Pearson correlation (95% CI) 0.78 (− 0.08–0.98) with *P* = 0.06 for mean ED LOS and mean visits per day by phase and Pearson correlation (95% CI) 0.71 (− 0.24–0.97) with *P* = 0.11 for mean ED LOS and proportion of imaging performed by phase.

## Discussion

In this retrospective, descriptive study we analyzed the developments at KH during a 30-day baseline period followed by the first 60 days of the COVID-19 outbreak in Stockholm Region, Sweden. Analysis of 9347 ED visits separated in six phases show that KH was able to improve ED crowding despite the challenges posed by COVID-19. ED crowding is a complex, multivariable issue and multiple factors may have impacted this development. To structure the analysis, we used the conceptual input, throughput, output model for ED crowding introduced by Asplin et al. [15] The main input factor that increased the demand for ED services was when patients with COVID-19 started to arrive in phase 2. The regional call-center with digital self-triage and a restrictive message to the public [18] starting in phase 2 together with the fear of acquiring COVID-19 when seeking care reduced the arrival intensity. In phase 4–6, ambulance diversion from other hospitals increased the pressure on the ED. Positive throughput factors were increased resources and higher competence in the ED [13, 20] that were added in phase 3 together with new working methods limiting diagnostics and aiming for quick clinical decisions to admit a higher proportion of patients to inpatient care [21, 22]. ED LOS was also statistically significantly lower in phase 3–6 compared to the phase 1–2 before the change. A key output factor was the reduced bed occupancy in the emergency wards that improved the service level for the ED. [23] This is in line with the findings of Asplin et al. that “the most frequently cited reason for ED crowding is the inability to move admitted patients from the ED to an inpatient bed” [15]. We also identified a statistically significant correlation between mean ED LOS and mean emergency ward occupancy by phase. The decrease in bed occupancy was enabled by a transformation of the inpatient care capacity in the hospital, increasing intensive care and infection ward capacity and shifting focus from specialized elective care to emergency care of COVID-19 patients. The transformation was enabled by an alignment of priorities across the hospital that was fueled by the general state of emergency and directed by the launch of the hospital command group in phase 2.

A key limitation was the study-design where the research group defined the cut-off points and the phases retrospectively. We aimed to define cut-off points as objectively as possible based on major events that impacted the conditions for the ED, but this may still have introduced bias. It was however not feasible to conduct a prospective intervention study as we were in a state of emergency, unable to predict how events would unfold and what measures that would be taken. Overall, the quality of the data is high as the study is based on standard reporting variables already existing in the data warehouse that is used daily. This is also a limitation as it narrows down the choice of proxy variables to use for the factors. One limitation is that ED LOS starts when the patient is registered and not when the patient arrives at the ED. When the arrival intensity is high, there may be additional waiting time before being registered. These situations are likely to be associated with ED crowding and extended ED LOS so high levels of ED LOS may be underestimated. Another limitation is that the number of staffed beds is based on manual input from the wards as capacity changes. This often leads to small delays as the priority is to first change the care capacity and then to report it in the system. This may have led to an underestimation of the intensive care capacity in the middle of phase 3. Generalizability may to some aspects be limited as there were some favorable conditions at KH. The hospital had a relatively large part of the staff engaged in research, education and specialized elective care that could be redeployed during the crisis. KH also have a large operating theatre with many physicians and nurses specialized in anesthesiology and perioperative care that could quickly be retrained to work in intensive care. In addition to this, the new operating theatre with pre- and postoperative wards were just recently inaugurated and had excellent capabilities to be transformed into intensive care wards for COVID-19 patients.

## Conclusions

It is possible to avoid ED crowding, even during extreme and quickly changing conditions by leveraging previously known input, throughput and output factors. One key factor was the change in working methods in the ED with higher competence, less diagnostics and increased focus on rapid clinical admission decisions. Another important factor was the reduction in bed occupancy in emergency wards that enabled a timely admission to inpatient care.

## Data Availability

The datasets used and/or analysed during the current study are available from the corresponding author on reasonable request.

## Abbreviations

ED: Emergency department
ED LOS: Emergency department length of stay
CI: Confidence interval
KH: Huddinge site at Karolinska University Hospital
EMS: Emergency Medical Services (ambulance or helicopter)
